# Colon cancer cells secrete exosomes to promote self-proliferation by shortening mitosis duration and activation of STAT3 in a hypoxic environment

**DOI:** 10.1186/s13578-019-0325-8

**Published:** 2019-08-06

**Authors:** Ruixue Ren, Hua Sun, Cui Ma, Jiatao Liu, Hua Wang

**Affiliations:** Department of Oncology, The First Affiliated Hospital of Anhui Medical University, 218 Jixi Road, Hefei, 230022 Anhui China

**Keywords:** Exosomes, Hypoxia, Cell proliferation, Mitosis, STAT3

## Abstract

Colon-cancer-cell-derived exosomes (CDEs) are emerging mediators of tumorigenesis and serve as messengers of intercellular communication; however, whether the CDEs affect the proliferation of colon cancer cells themselves remains unknown. In the current study, the CDEs isolated from human colon cancer cell line SW480 and HCT116 showed a size range of 60–150 nm, typical bilayer-encapsulated vesicles, and expressed the exosomal markers CD81 and CD63. Incubation of SW480 cells with CDEs labelled with PKH67 fluorescent markers revealed that SW480 cells were able to absorb CDEs, which were mostly distributed around the nucleus. Hypoxic conditions promoted colon cancer cells to release a greater number of CDEs than normoxic conditions. MTT cell proliferation assay demonstrated CDEs promoted the proliferation of colon cancer cells in a time- and dose-dependent manner. Mechanistically, CDEs promoted colon cancer cell growth mainly through shortening mitosis duration. Meanwhile, the levels of phosphorylated STAT3 in colon cancer cells was up-regulated with the treatment of CDEs derived from hypoxic tumor cells. Our data suggests that colon cancer cells are able to promote self-growth through the secretion of exosomes, especially under hypoxic conditions, which shortens mitosis duration and activates STAT3.

## Introduction

As the third most common cancer in the world, colorectal cancer (CRC), especially metastatic CRC, has received great attention in recent years due to its high morbidity and mortality [1]. Similarly, the incidence and mortality of CRC in China have also increased significantly [2]. The occurrence and development of CRC may be related to many risk factors, such as smoking, obesity and red meat consumption, and the recurrence and metastasis of CRC is the main cause of death [3]. Despite the great progress in the treatment of CRC including surgery and combined radiation therapy and chemotherapy, the 5-year survival rate of CRC is still poor and the overall prognosis of CRC is still not optimistic [4]. Therefore, the molecular mechanisms for exploring its occurrence and development needed to be explored urgently.

Small extracellular vesicles (also known as exosomes) can be released by tumor cells to promote tumor progression, playing an important role in communicating between tumor cells and their microenvironment [5]. Exosomes are membrane-enclosed vesicles approximately 30–150 nm in diameter and are widely distributed in various body fluids. Colon cell-derived exosomes (CDEs) have been shown to modulate local and systemic tumor microenvironment by transferring bioactive factors, such as nucleic acids and proteins [6]. However, the specific composition of exosomes depends on their parental cell type, as well as environmental factors, such as hypoxia.

Tumor hypoxia is a common phenomenon in solid tumors and has been widely considered as one of the most fundamental tumor microenvironment stresses for solid tumors [7]. Tumor hypoxia not only causes the problem affecting therapeutic efforts but may also acts as a selective pressure promoting tumor aggressiveness [7, 8]. In a hypoxic microenvironment, tumor cells reshape their microenvironment to sustain survival and rapid growth [7, 9], this results in the continuous selection of CRCs that have acquired abilities to withstand these harsh hypoxic conditions and adapt to a hypoxic microenvironment, thereby enhancing tumor proliferation [5]. In addition, hypoxia has been shown to increase exosome secretion and cancer invasion in many types of tumors. Hypoxia-inducible factors promote the formation of microvesicles that stimulate tumor cancer invasion and metastasis [7, 10]. Exosomes secreted under hypoxia enhance invasiveness of prostate cancer cells by targeting adherens junction molecules [11]. Hypoxia-induced exosomes induce a more aggressive and chemoresistant ovarian cancer phenotype [12]. All these studies demonstrate an important role of hypoxia related exosomes in exacerbating malignant progression of tumors.

The dysregulation of normal cell cycle control is a hallmark of human cancer. A variety of tumor cell-derived exosomes can transmit miRNA and mRNA to regulate cell cycle [13–15]. For example, exosomes derived from human primed mesenchymal stem cells induce mitosis of themselves [16]. At present, mitosis has attracted widespread attention as a chemotherapeutic target, and numerous strategies for targeting the cancer cell cycle have been proposed. However, whether the colon cancer-derived exosomes (CDEs) regulate self-proliferation by altering colon cancer cell cycle remains unknown. In the current study, we examined the effects of the CDEs isolated from human colon cancer cell line SW480 and HCT116 on proliferation of these cell lines and explored underlying mechanisms were also explored.

## Materials and methods

### Cell lines and culture

The cell lines SW480 and HCT were purchased from the Cell Bank of the Chinese Academy of Sciences (Shanghai, China). These cell lines were immediately expanded and frozen so that they could be restarted every 10 generation from a frozen vial of the same batch of cells. The SW480 and HCT cells were cultured in DMEM supplemented with 10% fetal bovine serum (Gibco, Grand Island, NY, USA) and penicillin/streptomycin (100 IU/ml and 100 mg/ml, respectively; Beyotime Biotechnology, Jiangsu, China). Cells were maintained at 37 °C in a 5% CO_2_ atmosphere. These cells were cultured under normoxic condition (normoxic group) and hypoxia model condition (co-cultured with CoCl_2_ to mimic hypoxia condition). The cells that were cultured with 10-cm dish was used as exosomes collection and the cells were cultured with 96-well plates was used as a subsequent cell proliferation assay.

### Antibodies

Primary antibodies used in this study included rabbit anti-CD63 (System Biosciences, USA), rabbit anti-CD81 (System Biosciences, USA), rabbit anti-Calnexin (Cell Signaling, China), mouse anti-β-tubulin (Sigma), rabbit anti-phospho-Histone H3 (Ser10) (Milipore Sigma), rabbit anti-Phospho-Stat3 (Tyr705), rabbit anti-STAT3, rabbit anti-Cyclin D1, rabbit anti-Hes1 (Cell Signaling, China). Secondary antibodies used in this study were goat anti-rabbit or mouse HRP, donkey anti-rabbit or mouse Cy5, goat anti-rabbit or mouse Alexa 594, goat anti-human FITC or goat anti-rabbit or mouse Alexa 488 (Jackson Immuno Research, West grove, PA, USA).

### Purification of exosomes from cell culture supernatants

SW480 cells were plated on 10-cm dishes at a density of 1 × 10^6^ cells per dish in the culture media described above. After 48 h, culture media were discarded, cells were washed three times with PBS, and 5 ml of serum-free culture medium was added to each dish. After 48 h or 72 h’ cell culture in normoxic condition (Exo-normoxic) and hypoxia condition (Exo-hypoxic) (cells were incubated in the presence of 100 μM CoCl_2_ to mimic the hypoxia condition for 24 h), then the cell culture media were collected and filtered using a 0.22-µm filter and collected in new tubes. The supernatants were centrifuged at 3000×*g* for 15 min to remove cells and cell debris. Then, supernatants were transferred to sterile vessels and 1.2 ml of Exo Quick-TC was added. And mixed well by inverting or flicking the tube. After refrigerating overnight (usually 18 h), the Exo Quick-TC/biofluid mixture was centrifuged at 4000×*g* for 35 min to collect exosomes. The exosomal pellets were resuspended in 120 μl using sterile 1× PBS.

### PKH67 labeling of exosomes and exosomes uptake into recipient cells

SW480-derived exosomes were collected from 100 ml of culture medium (20 10-cm culture dishes were used) as described above. The 5 μg exosomes for PKH67 labeling. Exosomes were labeled using PKH67 Fluorescent Cell Linker kits (Sigma-Aldrich, St. Louis, MO) according to the manufacturer’s instructions, with minor modifications.

To examine the uptake of exosomes into recipient CRC cells, DMEM containing either PKH67-labeled exosomal solution or control solution was added to each well. Cells were cultured for 24 h at 37 °C in a normal atmosphere with 5% CO_2_. The slides were washed three times with D-PBS(−) and fixed with 3.7% formaldehyde solution at room temperature for 10 min. Slides were then washed three times in D-PBS(−). After the staining of nuclei using a Pro Long Gold Antifade Reagent with 4′,6-diamidino-2-phenylindole (DAPI; Life Technologies), the slides were covered with coverslips and visualized under a confocal laser scanning microscope (LSM710; Carl Zeiss, Oberkochen, Germany).

### Immunofluorescence

Cells plated on coverslips were pre-extracted with 0.2% Triton X-100 in PHEM for 45 s before fixation with 4% paraformaldehyde in PBS. After staining, experiments for CRC cells were fixed directly in 4% paraformaldehyde before extraction. Then, cells were blocked with 1% bovine serum albumin in TBST for 30 min, incubated with primary antibodies for 2 h at room temperature, washed with TBST three times and incubated with secondary antibodies for an additional 1 h at room temperature. DNA was stained with 4,6-diamidino-2-phenylindole for 2–3 min. Images were acquired using a DeltaVision microscope (GE Healthcare, Buckinghamshire, UK).

### Western blotting

The exosomes isolated by Exoquick™ precipitation or colon cancer cells were lysed with RIPA buffer (Sigma, USA). The protein concentration of lysates using the Bradford Assay Kit (Abcam, USA) with Thermo Scientific™ NanoDrop™ One (USA). Samples were subjected to SDS-PAGE on 12% tris–glycine gels and blotted onto nitrocellulose membranes. Membranes were probed with specific primary antibodies over-night at 4 °C followed secondary antibody for 1 h at room temperature and visualized by the ECL detection system.

### Live-cell imaging

For live-cell imaging, cells on coverslips were mounted in Rose chambers and maintained at 37 °C in phenol-free L-15 medium (Invitrogen) with 10% fetal bovine serum. Time-lapse images were acquired at 3 min intervals with a 100 × 1.4 NA PlanApo objective lens mounted on an Eclipse Ti microscope (Nikon, Tokyo, Japan). Z-stacks were collected at 1-μm steps.

### Electron microscopy

The purified CDEs were treated with RNase A to degrade any non-CDEs RNA. SW480 Exo-normoxic and Exo-hypoxic were analyzed for the size of particles and morphology by transmission electron microscope (TEM). Exosomes were suspended in glutaraldehyde, and ~ 3–5 µl of exosomes were applied to 400 mesh copper grids (formvar/carbon coated, glow-discharged) for 5 min, followed by negative staining with 2% uranyl acetate for 2 min. Grids were briefly washed in water, allowed to dry and viewed using FEI Technai Transmission electron microscope.

### MTT assay

SW480 and HCT116 cell lines (3–5 × 10^5^/ml) were cultured in 96-well plates and then treated with the required reagents in DMEM with 5% FBS. Then, the samples were exposed to hypoxia (use CoCl_2_ at the final concentration of 100 μM in our cell culture media to induce hypoxia). Add the CoCl_2_ containing media to our cells and incubate the cultures for 24 h in a conventional incubator (37 °C; 5% CO_2_) for 24 h. At the end of the incubation at 37  °C, the PAECs were incubated for another 4 h in a medium containing 0.5% 3-[4,5-dimethylthiazol-2-yl]-2,5-diphenyl-tetrazolium bromide (MTT). The reaction was terminated by the addition of DMSO to the medium. The absorbance at 540 nm was measured using a spectrophotometer.

### Statistical analysis

Data are expressed as the mean ± SEM and were analyzed using GraphPad Prism software (v. 5.0a; GraphPad Software, La Jolla, CA). To compare values obtained from two groups, the Student t test was performed. P values of < 0.05 were considered significant. Ted intensities of the full nucleus were measured by ImageJ. Data were from three or more independent experiments.

## Results

### The secretion of CDEs is increased when colon cancer cells are exposed to a hypoxic environment

**Fig. 1:**
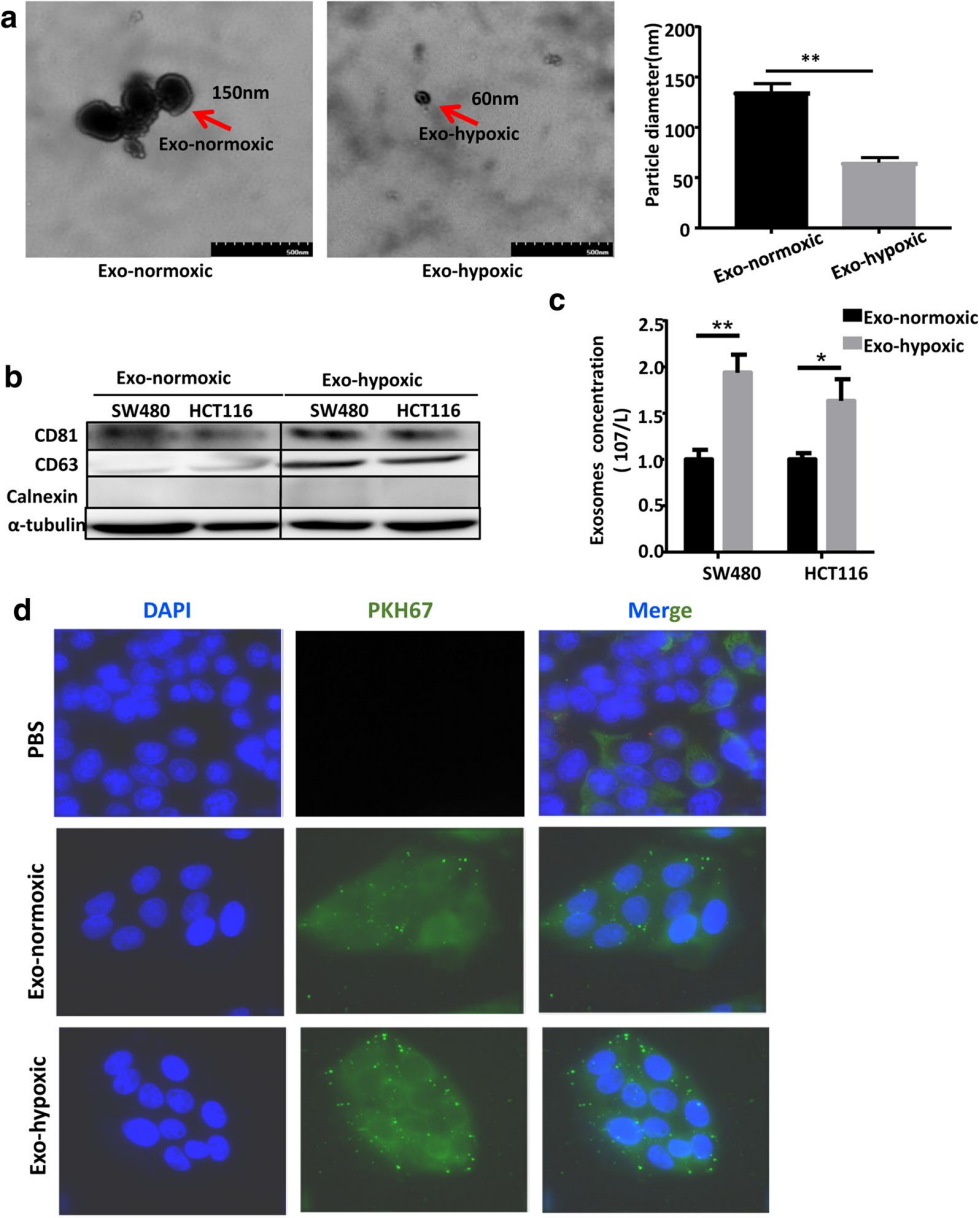
The secretion of CDEs is increased when colon cancer cells were exposed to a hypoxia environment. **a** Exo-normoxic and Exo-hypoxic were analyzed by electron microscopy and representative photomicrographs are shown (arrow points to exosomes, magnification, ×200,000, scale bar, 50 nm. The quantification of CDEs number in the picture on the right (n = 4). **b** Exo-normoxic and Exo-hypoxic collected through precipitation method were lysed and protein expressions of CD81, CD63, Calnexin, and α-tubulin were analyzed by Western blotting after equal amount of protein loading. **c** The number of exosomes released from SW480 and HCT116 cell line in normoxia and hypoxia respectively. **d** Immunofluorescence imaging analysis PKH67-labeled exosomes (Exo-hypoxic and Exo-normoxic) derived from the CRC cells were taken up by their own tumor cells (*P < 0.05; **P < 0.01)

As shown in Fig. 1a, TEM revealed the “cup-shaped” vesicles of CDEs, which is consistent with previous reports [17]. TEM results showed that the average size of Exo-normoxic (150 nm ± 5) was larger than Exo-hypoxic (60 nm ± 5). We also quantified the number of CDEs (4 samples were included in each group). Furthermore, we also characterized four exosomal protein markers of exosomes derived from two different CRC cell lines (SW480 and HCT116 cells) by western blotting analysis. The membrane protein calnexin is not expressed in CDEs, indicating that a high purity of CDEs. The CDEs marker proteins CD63 and CD81 were detected in both Exo-normoxic and Exo-hypoxic, however, the levels of CD63 and CD81 were much higher in Exo-hypoxic than conditions for both cell lines as shown in Fig. 1b. In addition, higher concentrations of CDEs were observed in the Exo-hypoxic conditions in both SW480 and HCT116 cells (Fig. 1c). These data indicated that hypoxic conditions induce more but smaller exosome release from CRC cells than normoxic conditions.

To examine whether CDEs can be absorbed by CRC cells, we labeled CDEs with PKH-67 green dye and incubated these PKH67-labeled CDEs (PKH-67 are lipophilic cell tracking dyes with long aliphatic tails into lipid regions of exactly lipophilic on exosomes CDEs) with SW480 cells for 12 h. As illustrated in Fig. 1d, CDEs from both Exo-normoxic and Exo-hypoxic conditions were significantly absorbed by SW480 cells. These observations suggested that exosomes derived from hypoxic CRC cells were more susceptible to be absorbed by CRC cells.

### Exo-hypoxic conditions promote more colon cancer cell line self-proliferation than Exo-normoxic conditions

**Fig. 2:**
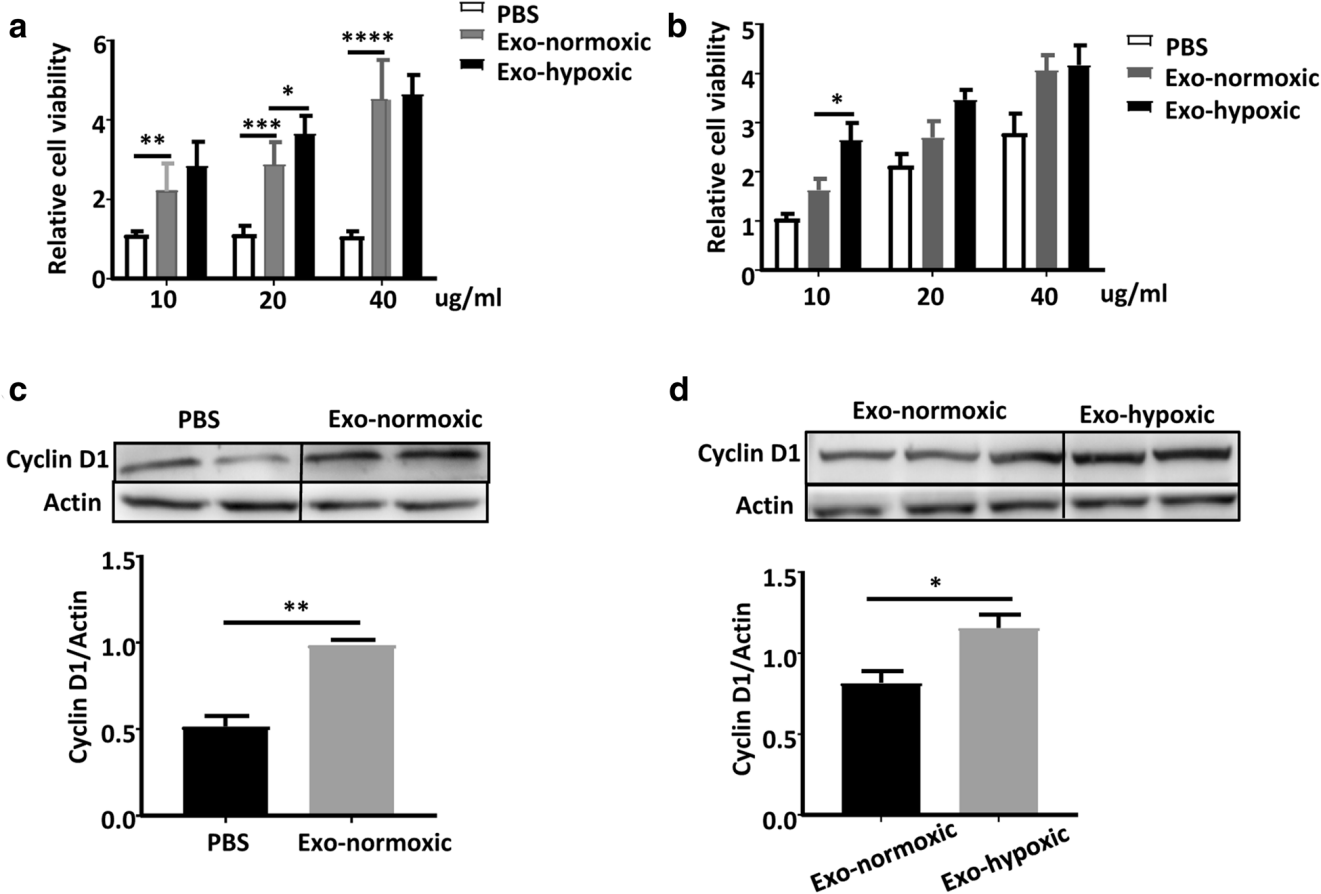
Exo-hypoxic conditions promote colon cancer cell line self-proliferation more significantly than Exo-normoxic. **a** Colon cancer cells SW480 were incubated with different concentrations of Exosomes, including 10 µg/ml, 20 µg/ml and 40 µg/ml, and cell viability was analyzed by MTT assays at 24 h. **b** Colon cancer cells HCT116 were treated with different concentrations of Exosomes, including 10 µg/ml, 20 µg/ml and 40 µg/ml, and cell viability was analyzed by MTT assays at 24 h. **c** Western blotting analysis of cyclin D1 expression in SW480 cells treated with the indicated concentrations of SW480‐derived exosomes for 24 h. **d** Western blotting analysis of cyclin D1 expression in SW480 cells treated with the indicated concentrations of Exo-normoxic and Exo-hypoxic for 24 h. Data are expressed as mean of three replicated experiments. Data are presented as the mean ± standard deviation (*P < 0.05; **P < 0.01; ***P < 0.001)

To examine the effects of CDEs on SW480 cell proliferation, we incubated SW480 cells with CDEs. As illustrated in Fig. 2a, incubation with Exo-normoxic or Exo-hypoxic CDEs (10, 20 and 40 μg/ml) for 24 h markedly increased the number of SW480 cells compared to PBS-treated group. Meanwhile, there was a higher number of SW480 cells with incubation of 20 µg/ml Exo-hypoxic than that with 20 µg/ml Exo-normoxic (Fig. 2a). Incubation with Exo-normoxic or Exo-hypoxic CDEs for 24 h also significantly increased the number of HCT116 cells compared to PBS-treated groups (Fig. 2b). There was a higher number of HCT116 cells with incubation of 10 µg/ml Exo-hypoxic than with 10 µg/ml Exo-normoxic (Fig. 2b).

We also checked the levels of cell cycle-related proteins, including cyclin D1 and Hes1. As illustrated in Fig. 2c, Exo-normoxic CDEs markedly increased cyclin D1 but not Hes1 expression in SW480 and HCT116 cells compared to PBS treatment. Additionally, incubation with Exo-hypoxic induced much higher levels of Cyclin D1 proteins in SW480 cells compared to incubation with Exo-normoxic (Fig. 2d).

### CDEs promote cell proliferation by reducing mitosis duration times of colon cancer cells

**Fig. 3:**
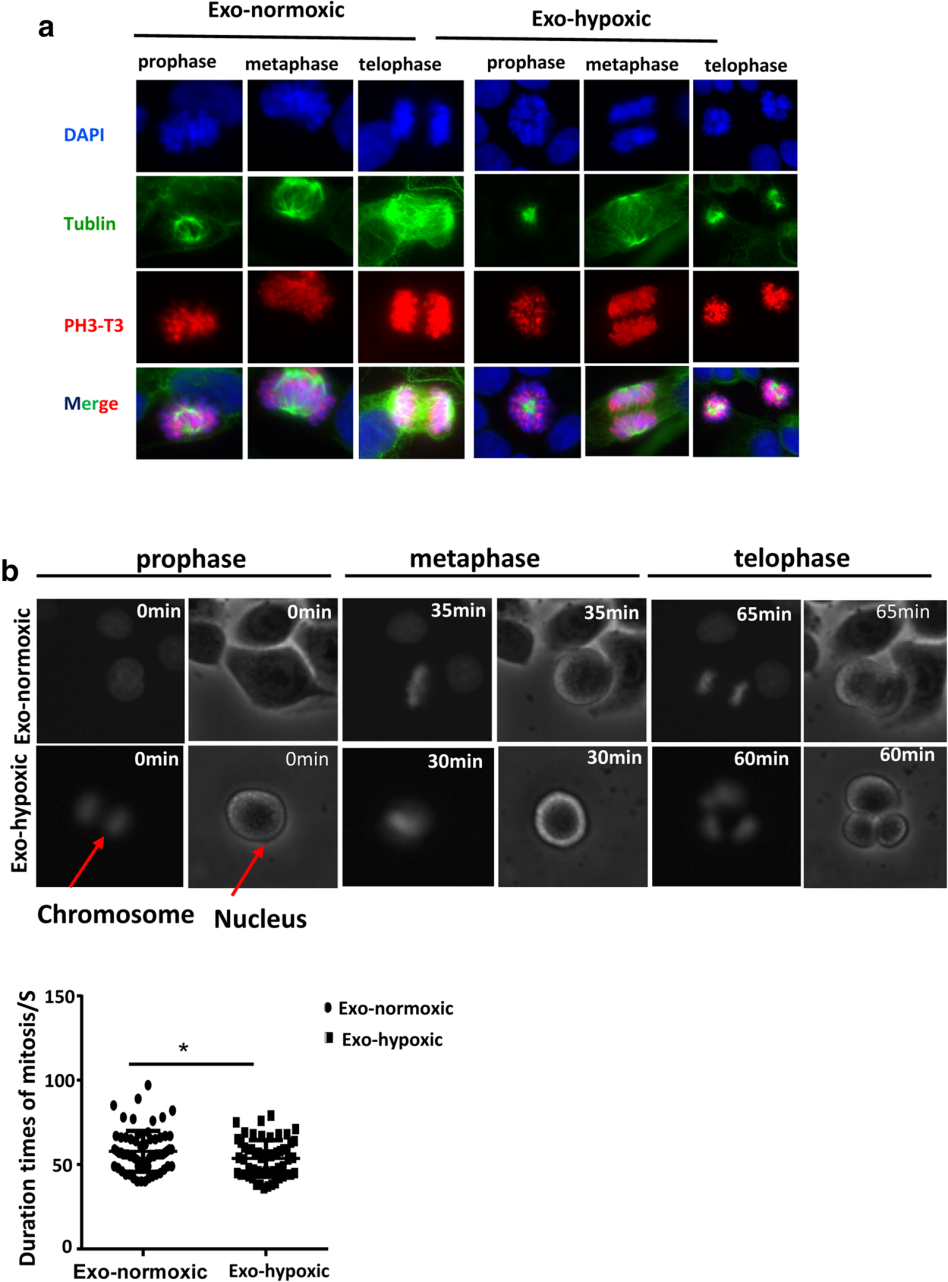
CDEs promote cell proliferation by shortening mitosis duration colon cancer cells. **a** Representative cell morphology of different stages of mitosis 24 h post the treatment of Exo-normoxic and Exo-hypoxic. **b** The changes of the morphology of nucleus and chromosome of tumor cells at different stages of mitosis were measured and the statistical results of duration mitosis were obtained (*P < 0.05)

To identify whether CDEs promote colon cancer cell proliferation by shortening the mitosis duration, SW480 cells were incubated with Exo-normoxic or Exo-hypoxic CDEs. During the experiment, live cell imaging was carried out. Morphological characteristics of chromosome and nuclei in different period of mitosis was observed by the confocal microscope and are shown in Fig. 3a, including prophase, metaphase, telophase. Our data revealed that the expression of mitotic marker phospho-histone H3 (pH3) was comparable between treatment with Exo-normoxic and Exo-hypoxic CDEs. Interestingly, we observed that the changes of morphological characteristics of chromosome and nucleus in different periods of mitosis were not synchronized (Fig. 3b). Exo-hypoxic-treated SW480 cells started cell division earlier than Exo-normoxic-treated cells, suggesting that Exo-hypoxic promotes cell proliferation by shortening mitosis duration times of colon cancer cells (Fig. 3c).

### CDEs from Exo-hypoxic conditions induce higher levels of phosphorylated STAT3 in colon cancer cells than that from Exo-normoxic conditions

**Fig. 4:**
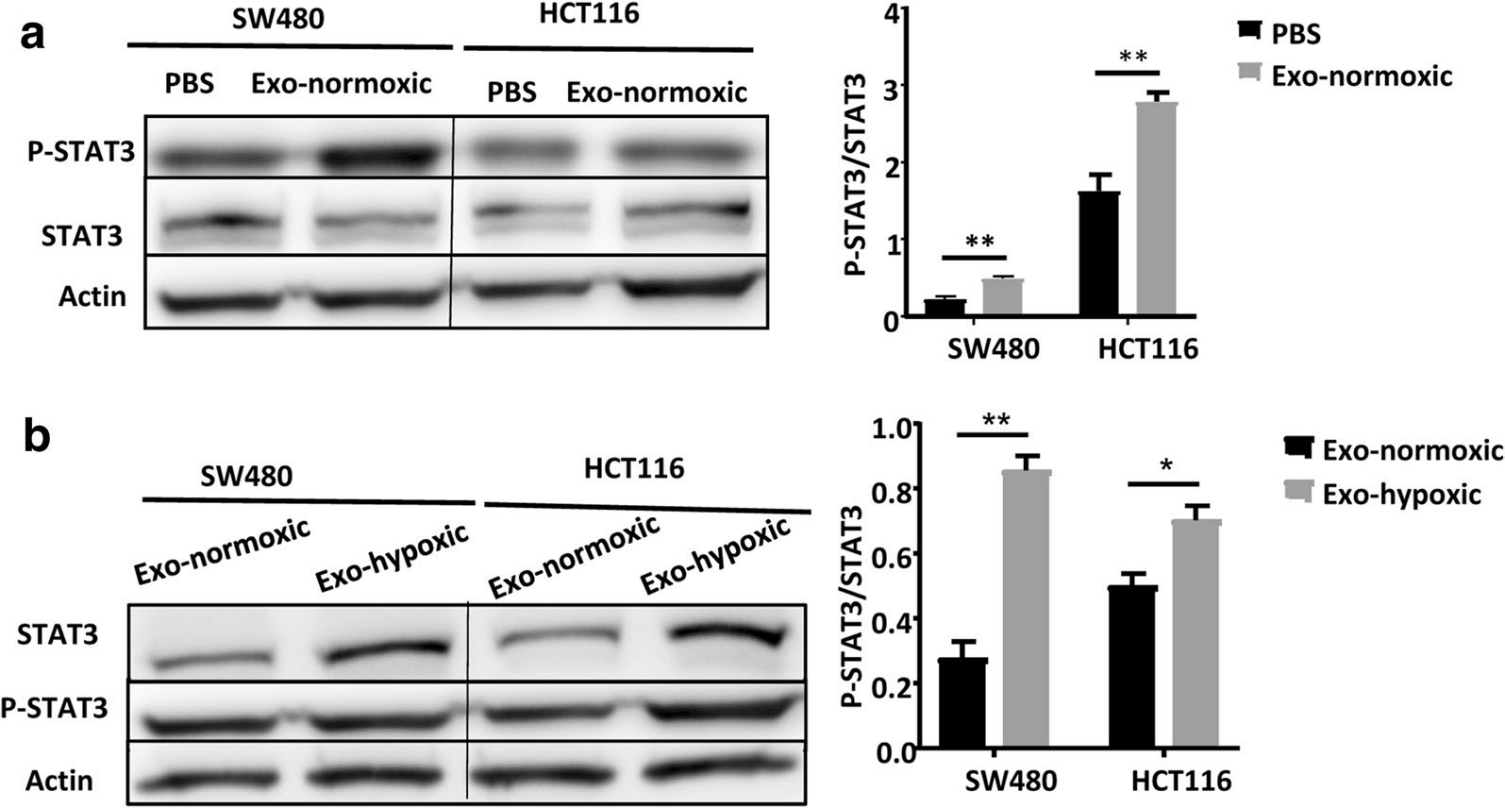
The phosphorylation of STAT3 is up-regulated after treatment of CDE exosomes. **a** Western blotting analysis of p-STAT3 expression of SW480 cells and HCT116 calls treated with PBS or Exo-normoxic for 24 h. **b** Western blotting analysis of pSTAT3 expression of SW480 cells and HCT116 calls treated with Exo-normoxic or Exo-hypoxic for 24 h. Data are presented as the mean ± standard deviation (*P < 0.05; **P < 0.01; ***P < 0.001)

As reported previously, persistent STAT3 activation in colon cancer is associated with enhanced cell proliferation and tumor growth [18], and tumor derived exosomes have been shown to activate the phosphorylation of STAT3 in ovarian cancer cells [12]. Thus, we wondered whether CDEs are able to activate STAT3 in CRC cells. As illustrated in Fig. 4a, incubation with CDEs from Exo-normoxic conditions induced greater STAT3 activation in both SW480 and HCT116 cells than PBS treatment. Moreover, Exo-hypoxic treatment induced higher levels of pSTAT3 in SW480 and HCT116 cells than Exo-normoxic treatment (Fig. 4b).

## Discussion

In this study, we found that the secretion of CDEs were increased when CRCs was exposed to a hypoxic environment. Interestingly, CRCs were able to absorb more Exo-hypoxic than Exo-normoxic. Both Exo-hypoxic and Exo-normoxic promoted the CRC cell proliferation with greater proliferation induced by Exo-hypoxic than by Exo-normoxic. Finally, Exo-hypoxic treatment induced stronger STAT3 activation and shorter mitosis duration in colon cancer cells than Exo-normoxic.

Hypoxia is an important feature of the tumor microenvironment. Hypoxic tumors exhibit more aggressive phenotypes and are associated with poor patient outcome in a wide variety of cancers [7]. In those hypoxic tissue regions, cancer cells remodel their unfavorable micro-environment for tumor, which usually cause resistance to cancer therapy [19]. Exosomes are vital mediators of intercellular communication that can transfer the hypoxic cells’ phenotype to non-hypoxic cells through the production of exosomes [17, 20]. Increasing studies have indicated that hypoxia promotes exosome secretion in various types of tumors [6], which may influence tumor initiation, growth and progression [7]. Multiple proteins or RNAs in cancer cells are involved in the release process of exosomes. Especially, the small GTPases, RAB27A and RAB27B, were participated in exosome secretion in various human tumor cells [21, 22]. In addition, there are reports showing that heat shock proteins (HSP70 and HSP90) were increased in exosomes due to cellular stresses [23]. These proteins inhibit cell apoptosis and increase cell proliferation so these exosomes provide a strong stimulus to the microenvironment that can facilitate the growth of cancers. In our study, we also found that the secretion of CDEs was markedly increased when CRCs were exposed to a hypoxia environment. However, the underlying molecular mechanisms remain unknown. More studies are required to be done to elucidate the mechanisms involved in the regulation of the number and size of exosomes secreted by CRC cells under different culture conditions.

It has been reported that hypoxia can promote the proliferation of CRCs, however, the mechanisms that hypoxia influence tumorigenesis are not fully understood. Exosomes are small microvesicles released from many types of cells and act as carriers of molecular information of cell-to-cell communication, transferring cargo content involved in many processes from parent to recipient cells. Exosomes provide the ability to transmit messages between cells at a distance and their roles in long distance communication have been well established [24]. The discovery of functional, transportable mRNA and miRNA within exosomes further increases the complexity of cell-to-cell communication. They can fuse with the recipient cells and deliver their contents into the cytoplasm of the recipient cell and perturb the recipient cell, especially since miRNA can mediate RNA interference [25]. They can also carry a combination of ligands to engage several cellular receptors at once modulating changes in the recipient cell [26]. CRC may modulate their own growth via autocrine signals provided by exosomes. The promotion roles of tumor cell proliferation by exosomes from colon cancer has been widely reported. For example, autocrine signals mediated by exosomes from the non-small cell lung cancer cell lines, glioma cells and gastric cancer cell lines improve cellular proliferation by increasing phosphorylation of Akt and extracellular signal regulated kinase [27]. The promotion roles of tumor cell proliferation by exosomes from colon [28] cancer has been reported in many studies. The contents carried by exosomes (including nucleic acids and proteins) have been confirmed as the cause for enhanced proliferation of CRCs. For example, ΔNp73 mRNA is enriched in CDEs. Incubation with CDEs containing ΔNp73 significantly increases the proliferative potential of target cells by inhibiting the function of tumor suppressor gene P53 [28, 29]. CRC Wnt5b-associated exosomes promote cancer cell migration and proliferation by inducing matrix metallopeptidases (MMPs) [30]. Also, MVP-mediated exosomal sorting of miR-193a promotes colon cancer progression through targeting of Caprin1, which upregulates Ccnd2 and c-Myc [14]. Our results revealed that Exo-hypoxic and Exo-normoxic significantly promote the proliferation of CRCs with greater proliferation induced by Exo-hypoxic than by Exo-normoxic, which is probably because Exo-hypoxic induce stronger STAT3 activation and shorter mitosis duration in colon cancer cells than normoxic as demonstrated in the current studies.

Chromatin instability is a major factor in the formation of tumors and shortening the length of mitosis can accelerate tumor cell proliferation [31, 32]. In our studies, we observed the localization and morphological changes of chromosomes of CRCs in various phases of mitosis after treatment with CDEs isolated from CRCs in Exo-hypoxic and Exo-normoxic condition. However, the cell mitosis duration was significantly shorter after treatment with Exo-hypoxic than with Exo-normoxic. Given that CDEs released from tumor cells carry oncogenic proteins that can alter the signaling pathways in the surrounding recipient tumor cells or normal cells in the hypoxic microenvironment [33], these oncogenic proteins may contribute to the regulation of mitosis duration. We also observed that greater STAT3 activation was observed after treatment with CDEs isolated CRCs in Exo-hypoxic than that with Exo-normoxic conditions. Activation of STAT3 has been shown to promote proliferation of colon cancer cell lines [34, 35]. Therefore, stronger STAT3 activation likely contributes to stronger CRC proliferation induced by Exo-hypoxic than by Exo-normoxic. In summary, CDCs from colon cancer cells can promote proliferation of themselves by shortening mitosis duration and activating STAT3. Thus, CDCs could be a novel therapeutic target for the treatment of colon cancer.

## Conclusion

In summary, increased secretion of CDEs by CRCs were observed in a hypoxic environment. The Exo-hypoxic promoted the CRC cell proliferation greater than by Exo-normoxic which was associated with stronger STAT3 activation and shorter mitosis duration in CRCs.

## Data Availability

All data generated or analyzed during this study are included in this published article.
